# A Rare Case of a Rectal Gastrointestinal Stromal Tumor (GIST) Discovered During a Routine Colonoscopy

**DOI:** 10.7759/cureus.41030

**Published:** 2023-06-27

**Authors:** Youssef Ghobrial, Rasiq Zackria, Sukhjinder Chauhan, Matthew Brockway, Pranati Shah, Mehrdad Asgeri

**Affiliations:** 1 Internal Medicine, MountainView Hospital, Las Vegas, USA; 2 Gastroenterology and Hepatology, Sunrise Health Graduate Medical Education (GME) Consortium, MountainView Hospital, Las Vegas, USA; 3 Internal Medicine, Touro College of Osteopathic Medicine, Las Vegas, USA; 4 Gastroenterology, Desert Regional Medical Center, Palm Springs, USA

**Keywords:** interstitial cells of cajal, cajal cell, c-kit mutation, rectal gist, cd 117 expression, cd34+, colonoscopy, gastrointestinal stromal tumor (gist)

## Abstract

A gastrointestinal stromal tumor (GIST) is a rare malignancy, accounting for only 0.1% to 3% of all gastrointestinal (GI) malignancies. Although GISTs are the most common mesenchymal tumor of the GI tract, they are primarily found within the stomach, with rectal GISTs rarely reported. They may present with rectal bleeding, constipation, pain, or a palpable mass while some are found incidentally. The incidence of GISTs has been on the rise, possibly due to advancements in diagnostic technology.

In this case report, we present a 50-year-old female who presented with intermittent constipation and rectal pain and was found to have a submucosal rectal mass during a routine diagnostic colonoscopy. Further evaluation confirmed the presence of a spindle-cell neoplasm, which was mildly cellular and showed positive expression of CD34 and CD117 on immunohistochemistry, consistent with the diagnosis of GIST of the rectum. This case report emphasizes the importance of routine colonoscopies in the early detection of neoplastic lesions of the colon and highlights the rare incidence of GISTs, their risk factors, pathogenesis, and common sites of occurrence.

## Introduction

Gastrointestinal stromal tumors (GISTs) are infrequently occurring neoplasms of the alimentary canal that are typically found in the stomach and small intestine. However, in rare cases, they may be located in the rectum. Rectal GISTs may be diagnosed incidentally or present with symptoms, including defecation problems, bleeding, and/or pain. Rectal GISTs have a higher risk of recurrence and have been associated with a poorer prognosis as compared to gastric GISTs.

Therefore, it is important to maintain a high index of suspicion for the diagnosis of these tumors. A multidisciplinary treatment approach and a long-term follow-up are recommended in patients with rectal GISTs.

## Case presentation

A 50-year-old woman with no significant medical history presented to the GI clinic for evaluation of intermittent constipation and rectal pain for the past year. The patient reported the use of over-the-counter laxatives to aid with bowel movements but occasionally had to perform manual disimpaction. During the digital disimpaction, she reported a sensation of two-to-three palpable masses in the anal canal. In addition, she reported associated rectal pain with occasional bright blood per rectum after disimpaction; otherwise, she denied any other symptoms. She reported a family history of colon cancer in her father at the age of 77. She also denied a prior colonoscopy.

**Figure 1:**
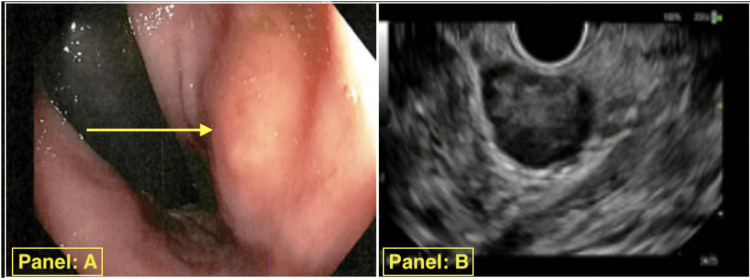
Routine colonoscopy revealed a 25 mm x 13 mm x 13 mm submucosal rectal mass (left panel A), confirmed by endoscopic ultrasound as a hypoechoic, heterogeneous lesion originating from the muscularis propria (right panel B)

During the routine colonoscopy, a submucosal mass was identified on the lateral aspect of the rectal wall about 2 cm from the anal verge, measuring approximately 25 mm x 13 mm x 13 mm in dimensions, as shown in Figure 1 (left panel A). To further evaluate the lesion, an endoscopic ultrasound (EUS) was performed, revealing a hypoechoic and heterogeneous mass arising from the muscularis propria, as shown in Figure 1 (right panel B).

A core needle biopsy was performed and histopathological analysis of the specimen showed a mildly cellular spindle-cell neoplasm. On immunohistochemical analysis, the neoplastic cells showed positive expression for CD34 and CD 117. These findings were consistent with a diagnosis of GIST. A Ki-67 assay was also performed and showed a proliferation rate of less than 1%. The patient was referred to oncology and general surgery for further management. The patient was started on neoadjuvant chemotherapy followed by elective surgical resection of the mass. One year following surgical resection, the patient underwent sigmoidoscopy, which was unremarkable and without evidence of recurrence.

## Discussion

GISTs are rare neoplasms of the gastrointestinal tract that account for only about 0.1-3% of all gastrointestinal malignancies [1]. These tumors were previously misdiagnosed as leiomyomas, leiomyoblastomas, leiomyosarcomas, or schwannomas due to their similar appearance on light microscopy. However, the discovery of gain-of-function mutations in the c-KIT proto-oncogene in 1998 led to the recognition of GISTs as separate tumor entities [2-3]. Hence, since 2000, studies have reported an increase in the incidence of GISTs, which is likely due to improvements in diagnostic capabilities [4]. At present, the estimated annual incidence of GISTs is seven to 15 cases per million across the globe [5].

GISTs originate from the interstitial cells of Cajal, which are considered to be the pacemaker cells of the gastrointestinal tract (GIT) and are involved in the regulation of gut peristalsis. These cells express c-KIT (CD117), which is a type III tyrosine kinase receptor. Mutually exclusive mutations in the KIT proto-oncogene or platelet-derived growth factor receptor alpha (PDGFRA), resulting in constitutive activation of KIT signaling, have been implicated in the pathogenesis of these neoplasms [1-2]. GISTs are usually diagnosed later in life, with the median age of diagnosis being 65 years [6]. Males and females are equally affected [1]. According to a SEER (Surveillance, Epidemiology, and End Results) database analysis involving 7204 patients, the incidence rate of GISTs in African Americans was twice as high as compared to Caucasians [7]. Recent studies also suggest that in comparison to the general population, morbidly obese patients undergoing bariatric surgery have a considerably higher incidence of GISTs [8].

GISTs can be located anywhere in the GIT. The most common sites of occurrence include the stomach (60-70%) and the small intestine (25-30%). Less commonly, they can be found in the colon, rectum, mesenteries, omentum, or retroperitoneum. Rectal GISTs constitute around 5% of all GISTs and comprise approximately 0.1% of all rectal neoplasms [2]. The clinical presentation varies according to the tumor location and tumor size. In many patients, these tumors are detected incidentally. However, in some cases, patients may be symptomatic. For instance, patients with gastric GISTs may present with upper gastrointestinal bleeding (e.g., melena or acute hematemesis) while small intestinal GISTs are more likely to present with intestinal obstruction. Rectal GISTs may present as gastrointestinal bleeding, intestinal obstruction, abdominal pain, perforation, palpable pelvic mass, or defecation problems. In some cases, rectal GISTs may be detected incidentally as a palpable mass during a digital rectal examination, as in our patient [9].

The diagnostic workup of GISTs includes an endoscopic examination, which allows for the direct visualization of the tumor and can also be used to perform biopsies. Contrast-enhanced computed tomography (CT), magnetic resonance imaging (MRI), and EUS are commonly used imaging modalities for the diagnosis of GISTs. CT or MRI scans are excellent tools for diagnosing, staging, and surgical planning and have the ability to assess the size, shape, and borders of the tumor, along with the possibility of distant metastatic disease. Per Eisenberg BL et al, GISTs exhibit varying sizes, with a median size of approximately 8.9 cm in high-risk cases, and are characterized by a fleshy pink or tan-white appearance that can display signs of bleeding, central cystic degeneration, or necrosis [10]. EUS can demonstrate a tumor’s origin from the muscularis propria and help distinguish benign from malignant tumors. It can also help obtain guided biopsies. The definitive diagnosis is based on the histopathological characteristics of the tumor. Spindle cell type, epithelioid type, and mixed type are the three common morphological patterns demonstrated by GISTs. Immunohistochemistry is essential for the diagnosis. Characteristically, more than 95% of GISTs show positive staining for c-KIT (CD117). Other markers used for the diagnosis include DOG1, CD34, S-100 protein, smooth muscle actin (SMA), and Ki67 [5,11].

Surgical resection is the standard treatment for rectal GISTs. Early, lower rectal GISTs can be treated with local excision via a trans-anal, trans-sacral, or trans-vaginal approach. Neoadjuvant imatinib (tyrosine kinase inhibitor) therapy with surgery may be used for the treatment of locally advanced GISTs [2,12]. The prognosis of patients with GISTs depends on several characteristics, such as tumor size, mitotic count, and primary tumor site. Tumors >5 cm with >10 mitoses per high-power field (HPF) typically have a high rate of recurrence [1,13]. Similarly, GISTs arising from locations other than the stomach are associated with less favorable survival outcomes [14]. In particular, rectal GISTs have been found to have a high rate of local recurrence [2]. Therefore, long-term follow-up of patients with rectal GISTs is recommended after treatment. In general, imaging such as CT or MRI should be performed every three to six months for the first three to five years after treatment, followed by annual imaging [15].

In summary, rectal GISTs are extremely uncommon and can present with variable symptoms. Our patient, with a positive family history of colon cancer, was seen in the clinic for reported symptoms of intermittent constipation and rectal bleeding. The diagnosis of rectal GIST was established using colonoscopy, EUS, histopathology, and immunohistochemistry. The patient was treated with neoadjuvant chemotherapy and surgical resection.

## Conclusions

GISTs are rarely occurring neoplasms of the alimentary canal. These tumors are usually found in the stomach and small intestine. However, in rare cases, these may be located in the rectum. Rectal GISTs may be diagnosed incidentally or may present with defecation problems, bleeding, and/or pain. Rectal GISTs have a higher risk of recurrence and may be associated with a poorer prognosis as compared to gastric GISTs. Therefore, it is important to maintain a high index of suspicion for the diagnosis of these tumors. A multidisciplinary treatment approach and a long-term follow-up are recommended in patients with rectal GISTs.
